# Moving toward Individual Treatment Goals with Pegcetacoplan in Patients with PNH and Impaired Bone Marrow Function

**DOI:** 10.3390/ijms25168591

**Published:** 2024-08-06

**Authors:** Jeff Szer, Jens Panse, Austin Kulasekararaj, Monika Oliver, Bruno Fattizzo, Jun-ichi Nishimura, Regina Horneff, Johan Szamosi, Régis Peffault de Latour

**Affiliations:** 1 Department of Clinical Haematology, Peter MacCallum Cancer Centre, Royal Melbourne Hospital, Melbourne, VIC 3052, Australia; 2 Department of Oncology, Hematology, Hemostaseology and Stem Cell Transplantation, University Hospital RWTH Aachen, 52074 Aachen, Germany; 3 Centre for Integrated Oncology (CIO) Aachen-Bonn-Cologne-Düsseldorf (ABCD), 52074 Aachen, Germany; 4 Department of Haematological Medicine, King’s College Hospital, National Institute of Health Research/Wellcome King’s Clinical Research Facility, London SE5 9RS, UK; 5 Division of Hemtatology, Department of Apheresis Medicine, University of Alberta Hospital, University of Alberta, Edmonton, AB T6G 2B7, Canada; 6 Hematology Unit, Fondazione IRCCS Ca’ Granda Ospedale Maggiore Policlinico, 20122 Milan, Italy; 7 Department of Oncology and Hemato-Oncology, University of Milan, 20122 Milan, Italy; 8 Department of Hematology and Oncology, Osaka University Graduate School of Medicine, Osaka 565-0871, Japan; 9 Swedish Orphan Biovitrum AB, 171 65 Stockholm, Sweden; 10 French Reference Center for Aplastic Anemia and Paroxysmal Nocturnal Hemoglobinuria, 75010 Paris, France

**Keywords:** PNH, bone marrow dysfunction, haematological response, clinical response, pegcetacoplan

## Abstract

Paroxysmal nocturnal haemoglobinuria (PNH) is a rare, potentially life-threatening haematological disease characterised by chronic complement-mediated haemolysis with multiple clinical consequences that impair quality of life. This post hoc analysis assessed haematological and clinical responses to the first targeted complement C3 inhibitor pegcetacoplan in patients with PNH and impaired bone marrow function in the PEGASUS (NCT03500549) and PRINCE (NCT04085601) studies. For patients with impaired bone marrow function, defined herein as haemoglobin <10 g/dL and absolute neutrophil count <1.5 × 10^9^ cells/L, normalisation of the parameters may be difficult. Indeed, 20% and 43% had normalised haemoglobin in PEGASUS and PRINCE, respectively; 60% and 57% had normalised LDH, and 40% and 29% had normalised fatigue scores. A new set of parameters was applied using changes associated with clinically meaningful improvements, namely an increase in haemoglobin to ≥2 g/dL above baseline, decrease in LDH to ≤1.5× the upper limit of normal, and an increase in fatigue scores to ≥5 points above baseline. With these new parameters, 40% and 71% of PEGASUS and PRINCE patients had improved haemoglobin; 60% and 71% had an improvement in LDH, and 60% and 43% had an improvement in fatigue scores. Thus, even patients with impaired bone marrow function may achieve clinically meaningful improvements with pegcetacoplan.

## 1. Introduction

Paroxysmal nocturnal haemoglobinuria (PNH) is a rare and potentially life-threatening haematological disease characterised by complement-mediated intravascular haemolysis (IVH) and thrombosis [1]. PNH can be classified into one of three subtypes: classical PNH, PNH in the context of other primary bone marrow disorders, and subclinical PNH [2]. The clinical presentation of PNH can include anaemia with persistently low haemoglobin (Hb), and fatigue, both of which impair quality of life (QoL) [3]. Clinical manifestations of PNH are determined by the size of the PNH clone, the presence of bone marrow failure (BMF) [2], and the degree of IVH. Haematological parameters such as Hb levels and reticulocyte counts differ in patients with PNH, particularly in the setting of BMF syndromes [2]. Indeed, underlying BMF syndromes such as aplastic anaemia can contribute to haematopoietic stem cell clonal selection and subsequent clinical manifestations of PNH [3].

Standard treatment options for haemolytic PNH include complement inhibitors and supportive treatments [4]. Terminal complement C5 inhibition, with eculizumab and ravulizumab, has been demonstrated to decrease IVH and improve patient outcomes [5,6,7]. Responses to complement inhibition treatment in PNH patients can be heterogeneous [8,9]. Patients with BMF tend to have smaller PNH clones resulting in milder IVH, and thus patients may experience limited benefit from treatment with the C5 inhibitor eculizumab (Supplementary Figure S1) [2,9].

One limitation of C5 inhibitors is the potential for extravascular haemolysis (EVH), which is mediated by the proximal C3 complement protein. Since C5 inhibition does not block the early steps of the complement cascade, PNH erythrocytes are opsonised by the C3 cleavage product C3b, leading to phagocytosis in the liver and spleen [10,11]. Expert reviews have estimated that up to one third of C5 inhibitor-treated patients will develop EVH [12], although surveys of patients receiving at least 3 months of C5 inhibitor therapy suggest that anaemia and fatigue persist in an even greater proportion of patients [13].

Pegcetacoplan is a C3 inhibitor that provides broader control of haemolysis (both IVH and EVH) compared to C5 inhibitors (Supplementary Figure S1) [1,14]. The Phase 3 PEGASUS study demonstrated the efficacy of pegcetacoplan in patients with haemolytic PNH who were anaemic despite stable treatment with eculizumab [14]. Pegcetacoplan demonstrated clinically relevant improvements in lactate dehydrogenase (LDH), absolute reticulocyte count (ARC) and Functional Assessment of Chronic Illness Therapy (FACIT)-Fatigue scores and transfusion avoidance, and was superior to eculizumab in improving mean Hb level from baseline in patients with PNH at 16 weeks [14]. Furthermore, the improvements were sustained through 48 weeks of treatment [14,15]. Pegcetacoplan was also associated with sustained and clinically meaningful improvements in QoL scores, as measured by the European Organisation for Research and Treatment of Cancer (EORTC) QLQ-C30 [16].

The Phase 3 PRINCE study demonstrated the efficacy of pegcetacoplan in patients with PNH who were naïve to complement inhibitor therapy [11]. Pegcetacoplan was associated with significant improvements in Hb, LDH, ARC and FACIT-Fatigue scores compared to patients receiving supportive care, and more than 90% of patients were transfusion-free at 26 weeks. Pegcetacoplan was also associated with improvements in global health status/QoL score, as measured by the EORTC QLQ-C30 [11].

As new treatments for PNH emerge, clinical measures and haematological parameters beyond LDH should be considered when evaluating treatment success. Since C3 inhibition provides broader control of haemolysis, it is possible that a broader range of parameters are improved through treatment. Furthermore, as it may be difficult for patients with underlying diseases or comorbidities, including BMF, to achieve normalisation of these parameters, it could also be worthwhile evaluating such additional parameters for clinically meaningful improvements rather than normalisation.

Both PEGASUS and PRINCE studies excluded patients with overt BMF syndromes based on platelet, neutrophil and/or reticulocyte criteria. However, traditional BMF criteria are difficult to interpret in the context of haemolytic PNH. There is a subgroup of patients in the PEGASUS and PRINCE studies who, despite meeting the criteria for no overt BMF, had lower platelet, neutrophil and reticulocyte counts indicating impaired bone marrow function. This post hoc analysis assesses haematological and clinical response in patients with PNH who had impaired bone marrow function, treated with pegcetacoplan or eculizumab in the PEGASUS study, or with pegcetacoplan or supportive care (excluding previous treatment with complement inhibitors) in the PRINCE study. Expanding beyond normalisation, this study examines an increase from baseline values by at least 2 g/dL in Hb, LDH within 1.5 times the upper limit of normal (ULN), and an increase from baseline of at least 5 points in FACIT-Fatigue scores to evaluate treatment success in these patients.

## 2. Results

### 2.1. Patient Baseline Characteristics

In total, 80 patients (pegcetacoplan, n = 41; eculizumab, n = 39) were evaluated from PEGASUS, including 14 patients (5 and 9, respectively) with impaired bone marrow function, and 42 patients (pegcetacoplan, n = 35; control, n = 7) were evaluated from PRINCE, including 9 patients (7 and 2, respectively) with impaired bone marrow function. For patients in PRINCE with a decrease in Hb ≥ 2 g/dL from baseline or with a qualifying thromboembolic event secondary to PNH, there was an option to escape the control arm and start pegcetacoplan treatment. Patients in PRINCE who escaped the control group (n = 11) were not included in this analysis. Baseline demographic and clinical characteristics are described in Table 1. As expected, patients with impaired bone marrow function had lower reticulocyte, neutrophil, and platelet counts compared to patients without impaired bone marrow function [2]. Additionally, patients in the PRINCE study had markedly higher LDH levels at baseline, which is reflective of the disease burden in these patients who were naïve to complement inhibitor treatment. No specific trends in the distribution of PNH red blood cell clones were observed between treatment groups, or between patients with impaired bone marrow function vs. those patients with non-impaired bone marrow function. The percentage of red blood cells with partial (type II) or total (type III) loss of CD59 is helpful in managing anaemia, as haemolysis may contribute more significantly to anaemia in patients with type III cells [2]. In patients with a high proportion of type II cells, the partial expression of CD59 may protect from the majority of spontaneous haemolysis, with haemolysis mainly occurring during periods of enhanced complement activation, such as infection or trauma [2].

### 2.2. Normalisation of Haematological and Clinical Parameters

Overall, data confirmed that the proportion of patients with impaired bone marrow function reaching assessed levels of relevant haematological parameters, i.e., normalisation, was comparably lower than patients without impaired bone marrow function at week 16 in the PEGASUS study and week 26 in the PRINCE study (Table 2). The normalisation of Hb was reached by 20.0% and 42.9% of pegcetacoplan-treated patients with impaired bone marrow function in the PEGASUS and PRINCE studies, respectively, compared with 36.1% and 46.4%, respectively, in patients without impaired bone marrow function. Notably, no patient receiving eculizumab in the PEGASUS study or supportive care in the PRINCE study achieved the normalisation of Hb at week 16 or week 26, respectively. The normalisation of ARC was achieved by 60.0% of pegcetacoplan-treated patients with impaired bone marrow function in the PEGASUS study, and by 57.1% of pegcetacoplan-treated patients with impaired bone marrow function in the PRINCE study. In pegcetacoplan-treated patients without impaired bone marrow function, the normalisation of ARC was achieved by 77.8% in the PEGASUS study, and 60.7% in the PRINCE study. FACIT-Fatigue scores reaching at least the population norm were reported in 40.0% and 28.6% of pegcetacoplan-treated patients with impaired bone marrow function in the PEGASUS and PRINCE studies, respectively, compared with 50.0% and 64.3%, respectively, in patients without impaired bone marrow function. Among eculizumab-treated patients in the PEGASUS study, broadly similar trends were seen for greater improvements in patients without impaired bone marrow function compared to those with impaired bone marrow function. The small numbers of patients with impaired bone marrow function prevents formal statistical analysis; however, consistent with the primary findings from PEGASUS, haematological and clinical responses to eculizumab were generally worse than those seen after pegcetacoplan treatment across both patient strata.

**Figure 1:**
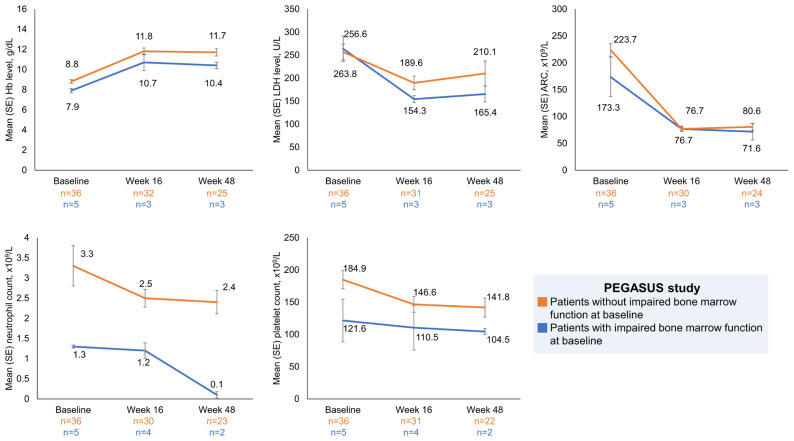
Mean haematological parameters over time for pegcetacoplan-treated PNH patients with and without impaired bone marrow function in the PEGASUS study. Patient numbers at week 16 and week 48 are as follows in the group of patients with impaired bone marrow function at baseline: haemoglobin, LDH and ARC, 3 patients each; platelets and neutrophils, 4 and 2 patients, respectively. Patient numbers at week 16 and week 48 are as follows in the group of patients without impaired bone marrow function at baseline: haemoglobin, 32 and 25 patients, respectively; LDH, 31 and 25 patients, respectively; ARC, 30 and 24 patients, respectively; platelets, 31 and 22 patients, respectively; neutrophils, 30 and 22 patients, respectively. ARC, absolute reticulocyte count; Hb, haemoglobin; LDH, lactate dehydrogenase; SE, standard error.

In the PEGASUS study, haematological parameters in patients with and without impaired bone marrow function were assessed over time (Hb, LDH, ARC, neutrophils, platelets) (Figure 1). Patients with impaired bone marrow function had a mean baseline Hb level of 7.9 g/dL, which improved to 10.7 g/dL at week 16 (mean Hb change from baseline [CFB] + 2.5 g/dL) and remained at 10.4 g/dL at week 48. Mean LDH levels decreased from a baseline value of 263.8 U/L to 154.3 U/L at week 16 (mean LDH CFB − 122.7 U/L), and 165.4 U/L at week 48. Mean ARC decreased from 173.3 × 10^9^ cells/L at baseline to 76.7 × 10^9^ cells/L at week 16 (mean ARC CFB − 115.6 × 10^9^ cells/L), and 71.6 × 10^9^ cells/L at week 48. Mean neutrophil counts were maintained from baseline to week 16 and decreased to 0.1 × 10^9^ cells/L at week 48. Mean platelet counts decreased from a baseline value of 121.6 × 10^9^ cells/L to 110.5 × 10^9^ cells/L at week 16, and 104.5 × 10^9^ cells/L at week 48. Changes in haematological parameters for patients with and without impaired bone marrow function are shown in Figure 1. FACIT-Fatigue scores increased in pegcetacoplan-treated patients with impaired bone marrow function by 7.0 points at week 16.

**Figure 2:**
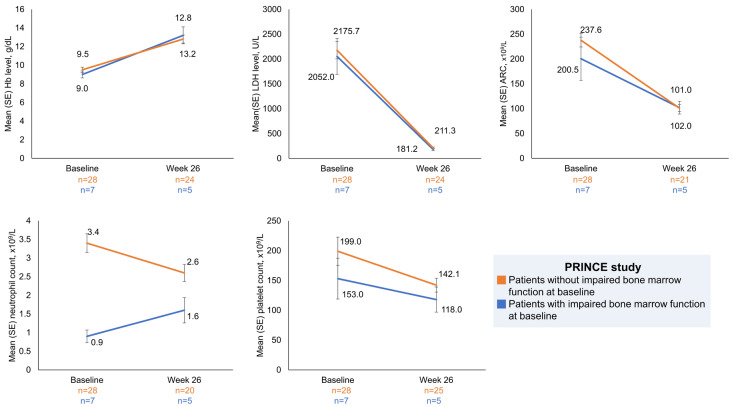
Mean haematological parameters over time for pegcetacoplan-treated PNH patients with and without impaired bone marrow function in the PRINCE study. Patient numbers at week 26 are as follows in the group of patients with impaired bone marrow function at baseline: haemoglobin, LDH, ARC, platelets, neutrophils, 5 patients each. Patient numbers at week 26 are as follows in the group of patients without impaired bone marrow function at baseline: haemoglobin, 24 patients; LDH, 24 patients; ARC, 21 patients; platelets, 25 patients; neutrophils, 20 patients. ARC, absolute reticulocyte count; Hb, haemoglobin; LDH, lactate dehydrogenase; SE, standard error.

Haematological parameters over time were also assessed in the PRINCE study from baseline to week 26 (Figure 2). Patients with impaired bone marrow function had a mean Hb level of 9.0 g/dL, which improved to 13.2 g/dL at week 26 (mean Hb CFB +3.9 g/dL). Mean LDH decreased from a baseline value of 2052.0 U/L to 181.2 U/L at week 26 (mean LDH CFB − 2306.3 U/L). Mean ARC decreased from a baseline value of 200.5 × 10^9^ cells/L to 102.0 × 10^9^ cells/L at week 26 (mean ARC CFB −148.7 × 10^9^ cells/L). Mean neutrophil counts increased from 0.9 × 10^9^ cells/L at baseline to 1.6 × 10^9^ cells/L at week 26. Mean platelet counts decreased from a baseline value of 153.0 × 10^9^ cells/L to 118.0 × 10^9^ cells/L at week 26. Changes in haematological parameters for patients with and without impaired bone marrow function are shown in Figure 2. FACIT-Fatigue scores increased in pegcetacoplan-treated patients with impaired bone marrow function by 6.0 points at week 26.

### 2.3. Assessment of Treatment Response Using Clinically Meaningful Changes as Target

**Figure 3:**
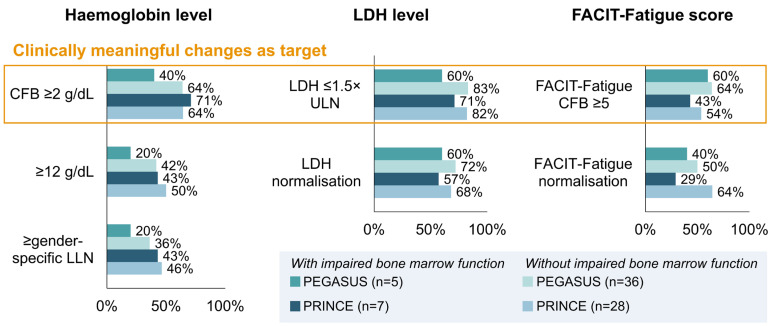
Proportion of pegcetacoplan-treated PNH patients with and without impaired bone marrow function reaching clinically meaningful treatment outcomes vs. normalisation. CFB, change from baseline; FACIT, Functional Assessment of Chronic Illness Therapy; LDH, lactate dehydrogenase; LLN, lower limit of normal; ULN, upper limit of normal.

Treatment response was then re-assessed for three parameters representing clinically meaningful changes in treatment outcomes for Hb, LDH and FACIT-Fatigue (Table 3). A higher proportion of PNH patients with impaired bone marrow function achieved the assessed targets compared to the normalisation of values (Figure 3). Overall, improvements in haematological and clinical parameters were seen in patients receiving pegcetacoplan treatment with or without impaired bone marrow function.

## 3. Discussion

The assessment and treatment of patients with PNH and impaired bone marrow function requires the evaluation of multiple clinical and laboratory factors. In patients treated with C5 inhibitors, anaemia may persist or recur as a consequence of residual IVH, or C3-mediated EVH [10]. These patients may experience improvements in LDH levels as a result of IVH control with C5 inhibition, but other abnormalities in haematological parameters may persist [10].

Pegcetacoplan offers additional treatment benefit over C5 inhibitors due to the additional targeting of EVH via the C3 complement blockade and therefore the ability to affect a broader range of parameters, even in patients with impaired bone marrow function. Patients with PNH and impaired bone marrow function showed benefits in haematological and clinical parameters in response to pegcetacoplan, in particular improvements in Hb, LDH, ARC, and FACIT-Fatigue scores at week 16 in PEGASUS and week 26 in PRINCE.

Compared to the overall study populations in PEGASUS and PRINCE, patients who had impaired bone marrow function showed a similar trend of increased Hb with pegcetacoplan treatment. Patients with impaired bone marrow function had a mean increase of 2.5 g/dL and 3.9 g/dL from baseline at week 16 and week 26, respectively, as compared with the overall study populations, with mean increases of 2.4 g/dL [14] and 2.9 g/dL [11] from baseline at week 16 and week 26, respectively. In the PEGASUS study, mean LDH decreased in pegcetacoplan-treated patients with impaired bone marrow function by 122.7 U/L by week 16, compared with a mean decrease of 15.0 U/L in the overall study population [14]. In the PRINCE study, mean LDH in pegcetacoplan-treated patients with impaired bone marrow function decreased by 2306.3 U/L by week 26, compared with a mean decrease of 1870.5 U/L in the overall study population [11]. This large decrease in LDH is not observed in patients in the control arm, who had a week 26 mean LDH level of 1535 U/L in the overall study population [11], highlighting the impact of C3 inhibition on IVH. Changes in ARC from baseline in pegcetacoplan-treated patients with impaired bone marrow function and the overall study populations were also similar. For patients in PEGASUS with impaired bone marrow function, mean ARC decreased by 115.6 × 10^9^ cells/L at week 16, compared with a mean decrease of 136 × 10^9^ cells/L in the overall PEGASUS population [14]. In the PRINCE study, patients with impaired bone marrow function had a mean decrease in ARC of 148.7 × 10^9^ cells/L, compared with the overall study population, which decreased by 123.3 × 10^9^ cells/L [11]. Mean FACIT-Fatigue scores were also comparable between patients with impaired bone marrow function and the overall study population. In PEGASUS, scores in pegcetacoplan-treated patients with impaired bone marrow function increased by 7.0 points at week 16, compared with 9.2 points [14] in the overall study population, and by 6.0 points compared with 7.8 points [11] in patients with impaired bone marrow function and the overall study population at week 26 in PRINCE, respectively.

Serum LDH level is a key parameter for evaluating PNH treatment success in current practice. However, it does not provide the full picture of a patient with PNH. For example, LDH may be significantly decreased or even normalised following the initiation of a C5 inhibitor, and yet anaemia may persist secondary to EVH or residual IVH. The new definition of haematological response to treatment in PNH [10] uses a broader range of parameters, including transfusion need, Hb level and ARC in addition to serum LDH. This post hoc analysis examines the use of additional haematological and clinical parameters, in particular FACIT-Fatigue as a measure of QoL, to evaluate treatment success in the era of proximal complement inhibition.

In a subset of PNH patients with impaired bone marrow function, the normalisation of haematological parameters by complement inhibition may not be feasible. Nevertheless, patients may still experience clinically meaningful improvements in symptoms. Defining individual treatment goals based on a patient’s individual disease characteristics and personal circumstances is becoming more common practice in several areas of medicine, including the management of type 2 diabetes [17] and cardiometabolic disease [18]. New targeted treatments for patients with PNH, such as the complement C3 inhibitor pegcetacoplan, have demonstrated significant and meaningful improvements in haematological and clinical parameters in patients with PNH. As these new treatments emerge, parameters beyond decreased LDH should also be considered when measuring treatment success. Treatment targets should consider the individual patient, assessing such aspects as lifestyle, work, and any underlying disease or comorbidity, and whether normalisation can be achieved.

As PNH is a rare disease, only a small number of patients are included in this analysis. The small sample size affects the reliability of the results due to increased variability. As such, outcomes may not be representative of the population data and conclusions should be made with caution.

## 4. Materials and Methods

### 4.1. Study Design and Population

**Figure 4:**
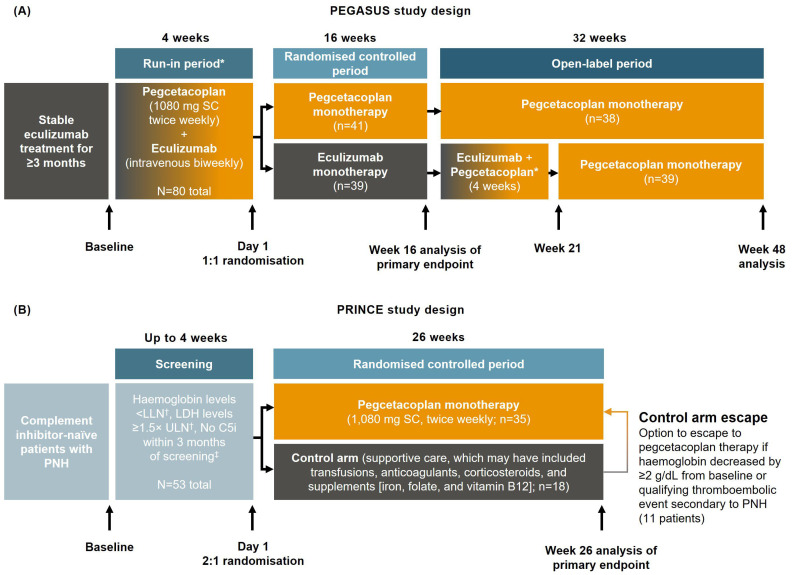
(**A**) PEGASUS [14,15] and (**B**) PRINCE [11] study designs. * Pegcetacoplan run-in periods in PEGASUS: (1) before randomisation, for both treatment groups; and (2) at start of open-label period, for the patients randomised to the eculizumab monotherapy group during the randomised controlled period. ^†^ Haemoglobin levels: LLN males, <13.6 g/dL; LLN females, <12.0 g/dL; LDH, 1.5 × ULN ≥339 U/L. ^‡^ All patients screened in PRINCE had never received a complement inhibitor at any point. C5i, complement 5 inhibitor; LDH, lactate dehydrogenase; LLN, lower limit of normal; PNH, paroxysmal nocturnal haemoglobinuria; SC, subcutaneous; ULN, upper limit of normal.

The study designs and patients included in the PEGASUS (ClinicalTrials.gov identifier NCT03500549; Figure 4A) [14,15] and PRINCE (ClinicalTrials.gov identifier NCT04085601; Figure 4B) [11] studies have been previously published.

This post hoc analysis of the PEGASUS and PRINCE studies evaluated the haematological and clinical response in patients with or without impaired bone marrow function.

### 4.2. Outcome Measures

Baseline characteristics and laboratory values for patients from PEGASUS and PRINCE with or without impaired bone marrow function were examined. Impaired bone marrow function was defined in this analysis as Hb level <10 g/dL and absolute neutrophil count <1.5 × 10^9^ cells/L [19]. All patients had platelet counts >50 × 10^9^ cells/L and neutrophil counts >0.5 × 10^9^ cells/L, per the study inclusion criteria [11,14].

Overall, haematological and clinical parameters were assessed at baseline and at weeks 16 and 48 for the PEGASUS study, and at week 26 for the PRINCE study. Normalisation of haematological parameters and clinical responses were evaluated at week 16 in PEGASUS and week 26 in PRINCE: Hb (gender-specific normal values; males: ≥13.6 g/dL; females: ≥12.0 g/dL) [14], Hb ≥12 g/dL [10], LDH (≤ULN: 226 U/L) [14], ARC (<ULN; males: 140 × 10^9^ cells/L; females: 120 × 10^9^ cells/L) [11], absolute neutrophil count (≥lower limit of normal (LLN): 1 × 10^9^ cells/L), platelet count (≥LLN: 140 × 10^9^ cells/L), transfusion avoidance, and FACIT-Fatigue score (≥population norm: 43.6) [20]. Haematological parameters were also assessed over time for the PEGASUS (baseline through week 48) and the PRINCE (baseline through week 26) studies.

Treatment success was subsequently evaluated at week 16 and at week 26 for the PEGASUS and PRINCE studies, respectively, using a new set of parameters. A CFB in Hb of 2 g/dL was selected, as this represents a clinically meaningful improvement in Hb [21]. The threshold for LDH (≤1.5 × ULN: 339 U/L) was selected, as values above 1.5 × ULN are associated with a significantly higher risk of thromboembolic events [22]. An improved FACIT-Fatigue score of CFB ≥5 points also represents a clinically meaningful improvement in fatigue [23].

Due to the small sample size and post hoc nature of the analysis, only descriptive statistics were used. No formal statistical testing was performed.

## 5. Conclusions

Patients with impaired bone marrow function may benefit from C3 inhibition, including those who had a suboptimal response to C5 inhibition. Across both PEGASUS and PRINCE studies, pegcetacoplan treatment was associated with improvements in haematological and clinical parameters regardless of bone marrow function status. While a smaller proportion of patients with impaired bone marrow function were able to achieve normalisation of Hb, LDH, ARC, neutrophil and platelet count, as well as FACIT-Fatigue scores, this is likely not treatment-related but rather due to the impaired bone marrow function. Using a new set of criteria, namely ≥2 g/dL CFB in Hb, LDH ≤1.5 × ULN, and FACIT-Fatigue ≥5 point CFB, a similar proportion of patients with and without impaired bone marrow function in the pegcetacoplan-treated groups of PEGASUS and PRINCE were able to achieve these clinically meaningful treatment outcomes. Therefore, considering indicators of a patient’s bone marrow function may be a more appropriate and beneficial approach to setting individualised treatment goals and evaluating PNH-targeted treatment success in the future.

## Figures and Tables

**Table 1:** Baseline characteristics, PEGASUS and PRINCE. ECU, eculizumab; FACIT, Functional Assessment of Chronic Illness Therapy; PEG, pegcetacoplan; PNH, paroxysmal nocturnal haemoglobinuria; SD, standard deviation.

	PEGASUS				PRINCE			
	PEG		ECU		PEG		Control	
Baseline Characteristics	Impaired Bone Marrow Function (N = 5)	Non-Impaired Bone Marrow Function (N = 36)	Impaired Bone Marrow Function (N = 9)	Non-Impaired Bone Marrow Function (N = 30)	Impaired Bone Marrow Function (N = 7)	Non-Impaired Bone Marrow Function (N = 28)	Impaired Bone Marrow Function (N = 2)	Non-Impaired Bone Marrow Function (N = 5)
Age, years (mean (range))	40.6 (19–81)	51.6 (22–79)	47.0 (30–71)	47.4 (23–78)	36.3 (29–45)	43.7 (22–67)	42.0 (28–56)	47.6 (20–67)
Female sex, n (%)	4 (80)	23 (63.9)	6 (66.7)	16 (53.3)	2 (28.6)	14 (50.0)	0 (0)	1 (20.0)
Mean body-mass index, kg/m^2^ (SD)	27.0 (7.50)	26.7 (3.87)	25.1 (5.25)	26.1 (4.00)	22.2 (5.42)	24.4 (4.13)	23.6 (0.53)	22.7 (2.35)
No transfusions within previous 12 months, n (%)	1 (20.0)	9 (25.0)	0 (0)	10 (33.3)	1 (14.3)	5 (17.9)	1 (50.0)	1 (20.0)
Duration of prior treatment with eculizumab, years (mean (range))	1.9 (1–3)	5.6 (0–17)	4.3 (0–14)	4.8 (0–14)	N/A	N/A	N/A	N/A
Eculizumab dose at screening, n (%)								
900 mg every 2 weeks	4 (80.0)	23 (63.9)	7 (77.8)	23 (76.7)	N/A	N/A	N/A	N/A
1200 mg every 2 weeks	0 (0)	12 (33.3)	2 (22.2)	7 (23.3)	N/A	N/A	N/A	N/A
1500 mg every 2 weeks	1 (20.0)	1 (2.8)	0 (0)	0 (0)	N/A	N/A	N/A	N/A
History of thrombosis, n (%)	3 (60.0)	12 (33.3)	4 (44.4)	6 (20.0)	2 (28.6)	(0)	0 (0)	0 (0)
Mean haemoglobin, g/dL (SD) *Normal reference range: males, 13.6*–*18; females, 12*–*16*	7.9 (0.50)	8.8 (1.09)	8.5 (0.73)	8.7 (0.93)	9.0 (0.97)	9.5 (1.49)	9.2 (0.89)	8.0 (0.83)
Mean lactate dehydrogenase, U/L (SD) *Normal reference range 113*–*226*	263.8 (61.07)	256.6 (102.30)	211.4 (38.12)	337.8 (319.53)	2052.0 (961.79)	2175.7 (912.54)	805.8 (248.55)	2183.7 (369.69)
Mean absolute reticulocyte count, ×10^9^/L (SD; median count) *Normal reference range: males, 10*–*140; females, 10*–*120*	173.3 (81.45; Median 155.0)	223.7 (73.12; Median 197.5)	184.6 (41.74; Median 193.3)	225.6 (73.36; Median 220.0)	200.5 (114.89; Median 190.0)	237.6 (70.99; Median 257.5)	90.0 (7.07; Median 90.0)	189.0 (39.91; Median 185.0)
Mean neutrophil count, ×10^9^/L (SD) *Normal reference range: 1*–*8*	1.3 (0.09)	3.3 (3.02)	1.0 (0.33)	2.8 (1.11)	0.9 (0.44)	3.4 (1.32)	1.0 (0.03)	2.5 (0.50)
Mean platelet count, ×10^9^/L (SD) *Normal reference range: 140*–*400*	121.6 (74.11)	184.9 (85.23)	116.3 (43.36)	165.9 (81.31)	153.0 (90.44)	199.0 (125.05)	76.0 (15.56)	142.4 (23.18)
Mean FACIT-Fatigue score (SD) *Population norm 43.6*	35.4 (12.32)	31.7 (11.36)	28.7 (10.26)	32.4 (13.17)	35.1 (11.14)	36.6 (10.73)	44.0 (4.24)	37.0 (11.62)
PNH red blood cells								
Mean %CD59 dim Type II, % (SD)	21.5 (26.29)	20.0 (19.15)	16.5 (11.33)	24.5 (19.29)	8.5 (13.75)	13.9 (18.68)	8.5 (6.66)	23.2 (29.47)
Mean %CD59 neg Type III, % (SD)	34.1 (14.83)	48.3 (21.98)	49.5 (20.71)	50.5 (22.10)	26.9 (26.88)	30.3 (16.39)	16.2 (12.66)	33.7 (14.85)
Mean %CD59 (dim Type II and neg Type III), % (SD)	55.6 (31.66)	68.4 (25.88)	65.9 (22.72)	75.0 (26.61)	35.3 (32.07)	44.2 (21.67)	24.7 (6.00)	56.9 (20.10)

**Table 2:** Normalisation of haematological and clinical response to treatment in patients with impaired bone marrow function. ARC, absolute reticulocyte count; ECU, eculizumab; FACIT, Functional Assessment of Chronic Illness Therapy; Hb, haemoglobin; LDH, lactate dehydrogenase; LLN, lower limit of normal; PEG, pegcetacoplan; ULN, upper limit of normal.

	PEGASUS Week 16				PRINCE Week 26			
	PEG		ECU		PEG		Control	
Haematological and Clinical Response Parameters, n (%)	Impaired Bone Marrow Function (N = 5)	Non-Impaired Bone Marrow Function (N = 36)	Impaired Bone Marrow Function (N = 9)	Non-Impaired Bone Marrow Function (N = 30)	Impaired Bone Marrow Function (N = 7)	Non-Impaired Bone Marrow Function (N = 28)	Impaired Bone Marrow Function (N = 2)	Non-Impaired Bone Marrow Function (N = 5)
Hb normalisation (≥gender-specific LLN: males, 13.6 g/dL; females, 12.0 g/dL)	1 (20.0)	13 (36.1)	0 (0)	0 (0)	3 (42.9)	13 (46.4)	0 (0)	0 (0)
Hb ≥ 12 g/dL	1 (20.0)	15 (41.7)	0 (0)	0 (0)	3 (42.9)	14 (50.0)	0 (0)	0 (0)
LDH normalisation (≤ULN [226 U/L])	3 (60.0)	26 (72.2)	6 (66.7)	17 (56.7)	4 (57.1)	19 (67.9)	0 (0)	0 (0)
ARC normalisation (<gender-specific ULN: males, 140 × 10^9^ cells/L; females, 120 × 10^9^ cells/L)	3 (60.0)	28 (77.8)	3 (33.3)	4 (13.3)	4 (57.1)	17 (60.7)	1 (50.0)	0 (0)
Neutrophil normalisation (≥LLN [1 × 10^9^ cells/L])	3 (60.0)	30 (83.3)	7 (77.8)	29 (96.7)	4 (57.1)	18 (64.3)	1 (50.0)	3 (60.0)
Platelet normalisation (≥LLN [140 × 10^9^ cells/L])	2 (40.0)	15 (41.7)	1 (11.1)	18 (60.0)	2 (28.6)	12 (42.9)	0 (0)	2 (40.0)
Transfusion avoidance	3 (60.0)	31 (86.1)	0 (0)	6 (20.0)	6 (85.7)	26 (92.9)	1 (50.0)	0 (0)
FACIT-Fatigue (≥population norm 43.6)	2 (40.0)	18 (50.0)	0 (0)	4 (13.3)	2 (28.6)	18 (64.3)	1 (50.0)	0 (0)

**Table 3:** Haematological and clinical response to treatment in patients with impaired bone marrow function according to clinically meaningful changes. CFB, change from baseline; ECU, eculizumab; FACIT, Functional Assessment of Chronic Illness Therapy; Hb, haemoglobin; LDH, lactate dehydrogenase; PEG, pegcetacoplan; ULN, upper limit of normal.

	PEGASUS Week 16				PRINCE Week 26			
	PEG		ECU		PEG		Control	
Haematological and Clinical Response Parameters, n (%)	Impaired Bone Marrow Function (N = 5)	Non-Impaired Bone Marrow Function (N = 36)	Impaired Bone Marrow Function (N = 9)	Non-Impaired Bone Marrow Function (N = 30)	Impaired Bone Marrow Function (N = 7)	Non-Impaired Bone Marrow Function (N = 28)	Impaired Bone Marrow Function (N = 2)	Non-Impaired Bone Marrow Function (N = 5)
Hb (CFB ≥ 2 g/dL)	2 (40.0)	23 (63.9)	0 (0)	0 (0)	5 (71.4)	18 (64.3)	0 (0)	0 (0)
LDH (≤1.5 × ULN [339 U/L])	3 (60.0)	30 (83.3)	8 (88.9)	23 (76.7)	5 (71.4)	23 (82.1)	0 (0)	0 (0)
FACIT-Fatigue (CFB ≥ 5)	3 (60.0)	23 (63.9)	2 (22.2)	6 (20.0)	3 (42.9)	15 (53.6)	1 (50.0)	0 (0)

## Data Availability

Data are not in a publicly available repository. Sobi is committed to responsible and ethical sharing of data on participant level and summary data for medicines and indications approved by EMA and/or FDA, while protecting individual participant integrity and compliance with applicable legislation. Data access will be granted in response to qualified research requests. All requests are evaluated by a cross-functional panel of experts within Sobi, and a decision on sharing will be based on the scientific merit and feasibility of the research proposal, maintenance of personal integrity, and commitment to publication of the results. To request access to study data, a data sharing request form (available at www.sobi.com, accessed on 1 August 2024) should be sent to medical.info@sobi.com. Further information on Sobi’s data sharing policy and process for requesting access can be found at: https://www.sobi.com/en/policies (accessed on 1 August 2024).

## Supplementary Materials

The following supporting information can be downloaded at: https://www.mdpi.com/article/10.3390/ijms25168591/s1.
