# Supplementary material for: Moving toward Individual Treatment Goals with Pegcetacoplan in Patients with PNH and Impaired Bone Marrow Function
Source: Int J Mol Sci. 2024 Aug 6;25(16):8591. doi: 10.3390/ijms25168591 (PMC11354612; doi:10.3390/ijms25168591)
Supplement: Supplementary file 1 [file ijms-25-08591-s001.zip › ijms-3105135-supplementary.pdf]

International Journal of
Molecular Sciences

MDPI

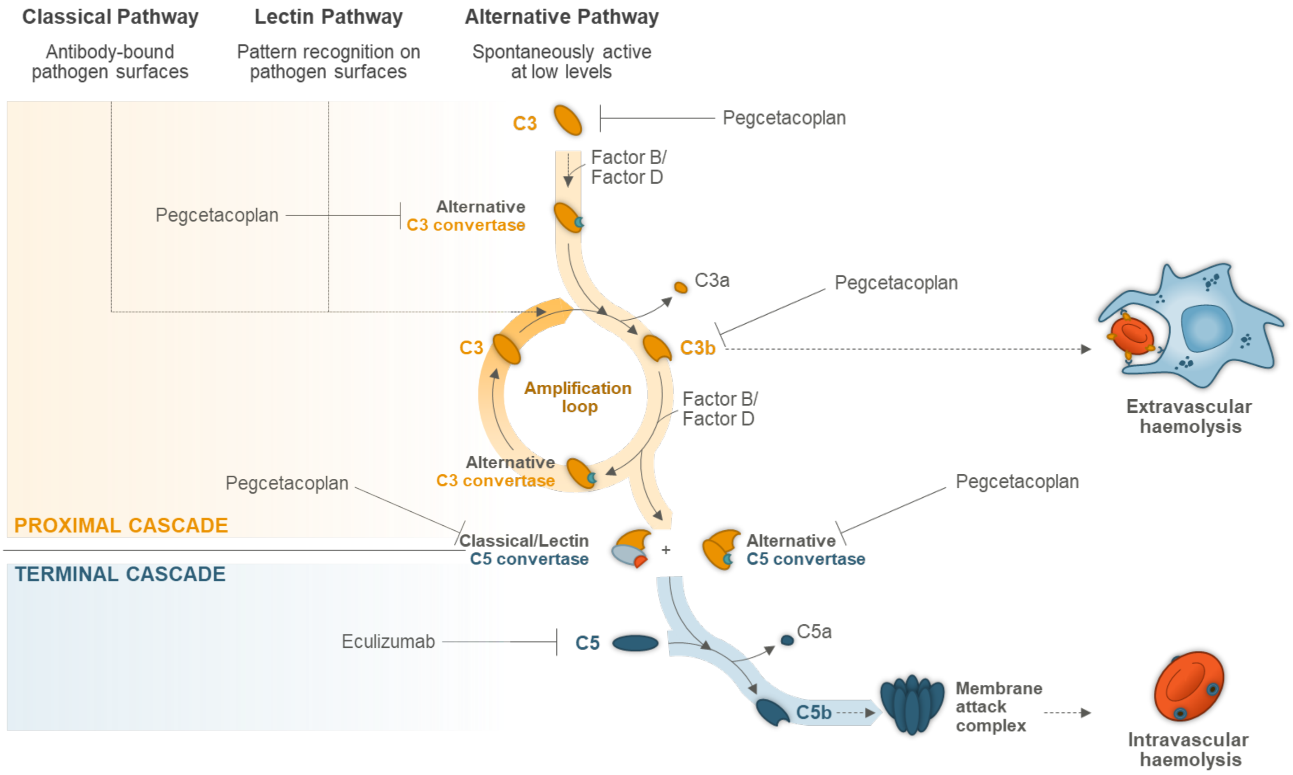

1

Supplementary Figure 1. Overview of the complement pathway and the mechanisms of action of pegcetacoplan and eculizumab. 2
